# Atypical Ocular Toxoplasmosis With Remote Vasculitis and Kyrieleis Plaques

**DOI:** 10.7759/cureus.52756

**Published:** 2024-01-22

**Authors:** Tan Teng Siew, Shahidatul-Adha Mohamad, Rafidah Sudarno, Haslinda Md Said

**Affiliations:** 1 Department of Ophthalmology and Visual Science, School of Medical Sciences, Universiti Sains Malaysia, Kubang Kerian, MYS; 2 Department of Ophthalmology, Hospital Tengku Ampuan Rahimah, Klang, MYS

**Keywords:** ocular toxoplasmosis, atypical, kyrieleis plaques, remote vasculitis, atypical ocular toxoplasmosis

## Abstract

Retinal vasculitis is common in ocular toxoplasmosis (OT) and typically occurs in the same quadrant as retinochoroiditis. This is a case of atypical ocular toxoplasmosis with remote vasculitis distant from the retinochoroiditis lesion. Examination of the left fundus showed the classic posterior segment finding of “headlight in the fog” in the absence of a chorioretinal scar. Retinal vasculitis was noted in all four quadrants at the periphery far from the retinitis area. A presumptive diagnosis of acute panuveitis secondary to ocular toxoplasmosis was made despite the enzyme-linked immunosorbent assay (ELISA) for Toxoplasmosis antibody being pending. The patient was treated empirically with oral sulfamethoxazole-trimethoprim for eight weeks and received both oral and topical corticosteroids. His symptoms and ocular signs have significantly improved. This case report highlights an atypical remote localization of vasculitis with the classic appearance of retinochoroiditis and vitritis, which is highly due to toxoplasmosis. Early initiation of antibiotic therapy is recommended despite pending serology to ensure a good final visual and ocular outcome.

## Introduction

Ocular toxoplasmosis (OT) is a common retinochoroiditis disease caused by the intracellular protozoan parasite, *Toxoplasma gondii *(*T. gondii*) [1]. *T. gondii *possesses the ability to infect mammals and birds, making it widely distributed across the world, affecting almost one-third of the world’s population [2,3]. About 1 in 400 people worldwide will develop OT, indicating that roughly 2% of toxoplasmosis patients will experience ocular symptoms [4].

OT is believed to arise either from the congenital or acquired route. In congenital infection, the fetus becomes infected through the placenta bloodstream, whereas, in acquired infection, the transfer of parasites usually occurs through the gastrointestinal tract.* T. gondii* infection in humans occurs through three primary pathways: 1) ingestion of contaminated food or water with *T. gondii* oocysts; 2) consumption of insufficiently cooked meat containing *T. gondii* tissue cysts; and 3) the passage of tachyzoites through the placenta during the initial maternal infection [2,5].

*T.gondii* consists of three different life forms: the oocyst, the tachyzoite, and the bradyzoite (tissue cyst). The feline or cat family is the definitive host for *T. gondii*, whereas humans and other mammals are intermediate hosts. Replication of oocysts occurs in cats’ intestines and they shed in the feces. Oocysts undergo sporulation and become infectious from 1-21 days in cat feces. When present in the environment, this oocyst contains sporozoites, which is a dormant form of *T. gondii* but is highly infectious when intermediate hosts ingest it, giving rise to tachyzoites. A tachyzoite is a rapid replication stage of the parasite once it penetrates all the nucleated cells and is disseminated throughout the tissues (brain, eye, muscle, and placenta) via blood streams in intermediate hosts. The bradyzoite remains dormant in the host’s tissues as a cyst (also known as a tissue cyst) and can become infectious once the host gets immunocompromised [2,5].

In this article, we describe a case of OT that presented with remote localization of vasculitis and Kyrieleis plaques, which is rare and atypically seen. Informed consent was taken from the patient’s parents for us to document this case report.

## Case presentation

**Figure 1:**
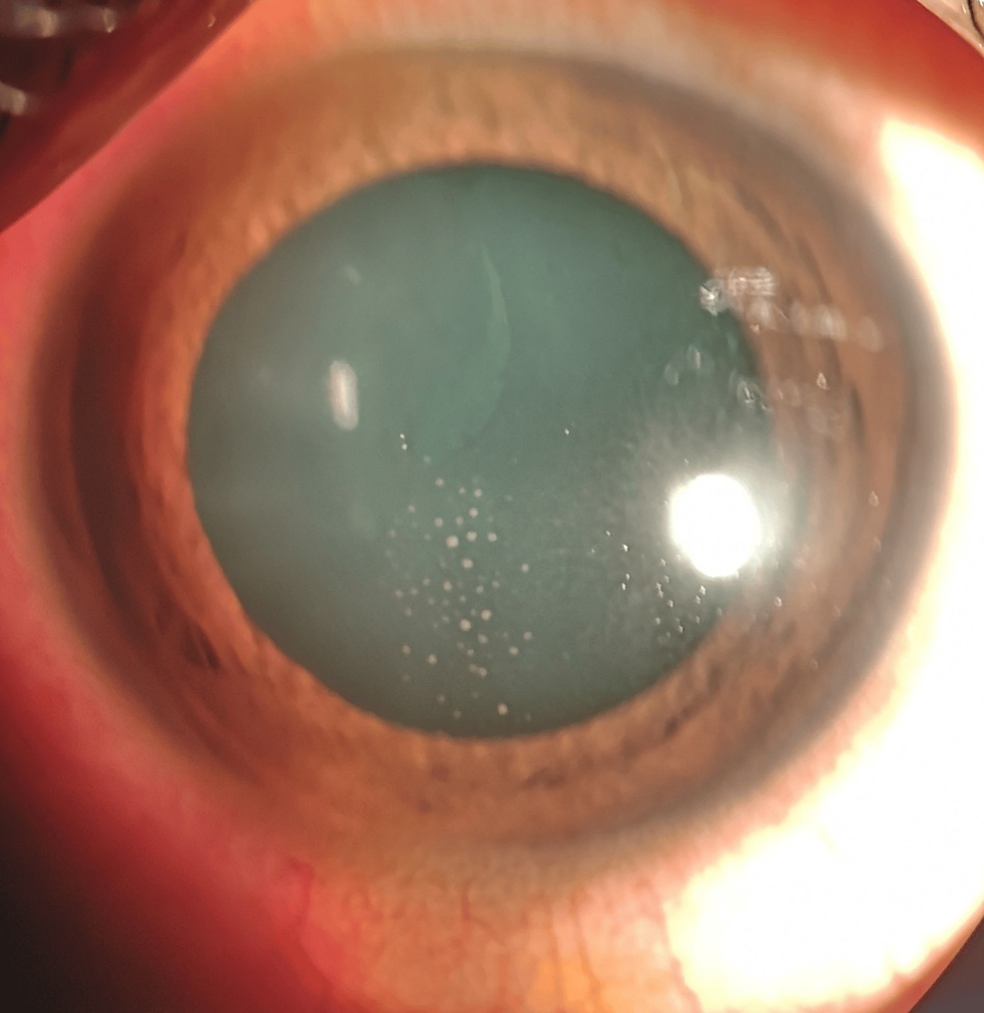
Keratic precipitates on the central corneal endothelium

A 12-year-old Malay boy with no known medical illness presented with merely left eye redness for three days without any other symptoms. His history was unremarkable except that he had 10 pet cats at home. His best-corrected visual acuity was 6/6 on the right eye and 6/9 on the left. No relative afferent pupillary defect (RAPD) was observed. The left eye showed mild conjunctival injection, granulomatous keratic precipitates on the central corneal endothelium (Figure 1), and the presence of anterior chamber cells 4+ with an intraocular pressure of 16 mmHg.

**Figure 2:**
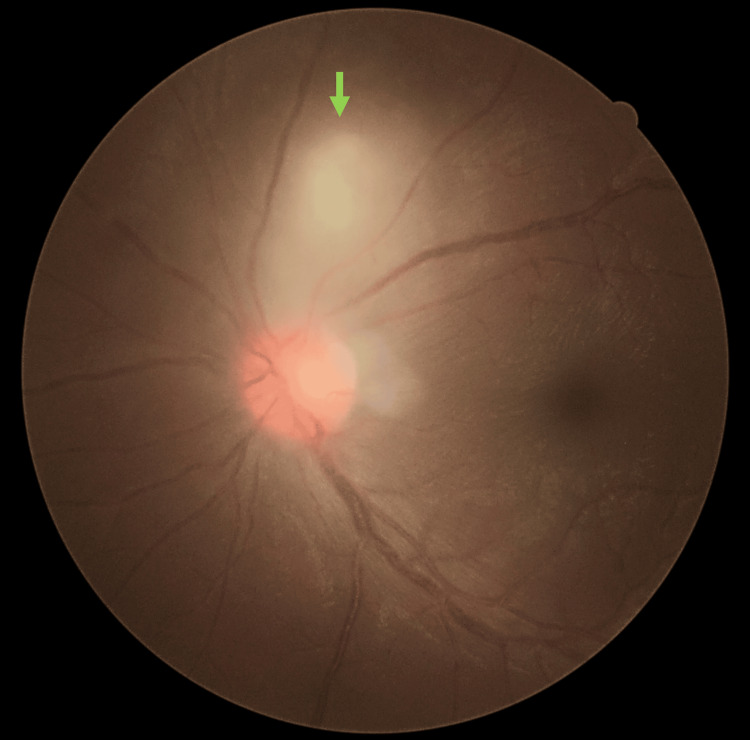
Classic “headlight in the fog” chorioretinitis (green arrow) was seen superior to the hyperaemic swollen optic disc during the presentation

Left fundus examinations noted a classic posterior segment finding of “headlight in the fog” focal chorioretinitis measuring 1 disc diameter (DD) size located 1 DD away from the optic disc superiorly (Figure 2). There was no chorioretinal scar adjacent to the focal chorioretinitis area. There were vitritis 1+, hyperemic swollen optic disc, mild macular striations, and multiple punctate choroiditis inferiorly.

**Figure 3:**
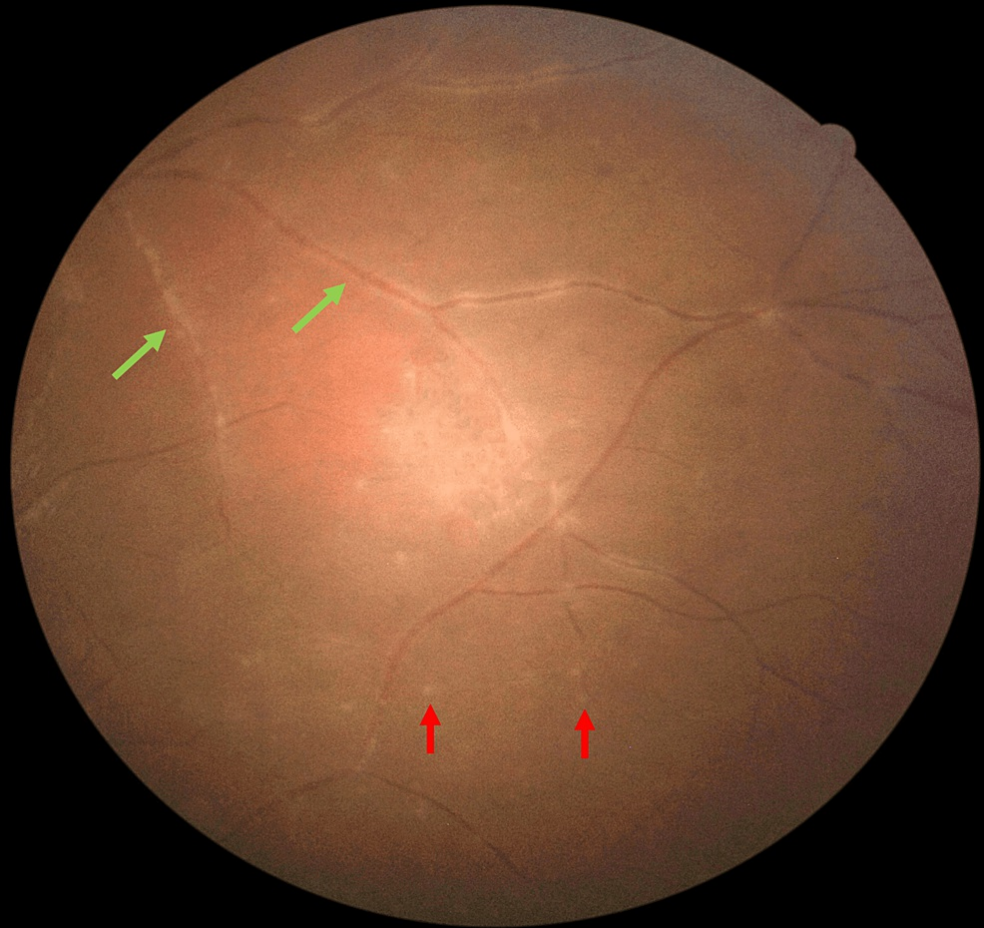
Fundus examination at presentation showed multiple punctate choroiditis (red arrows) with retinal vasculitis (green arrows) at the far periphery of the inferior retinal

There was no perivascular sheathing adjacent to the retinitis area, however, there was vasculitis in all four quadrants in the far peripheral retina (Figure 3). Right eye examinations were unremarkable. Optical coherence tomography (OCT) showed a left swollen optic disc but no evidence of any macular pathology. Systemic examinations were unremarkable. The clinical findings suggested that the possible differential diagnoses include ocular infections and autoimmune diseases.

**Figure 4:**
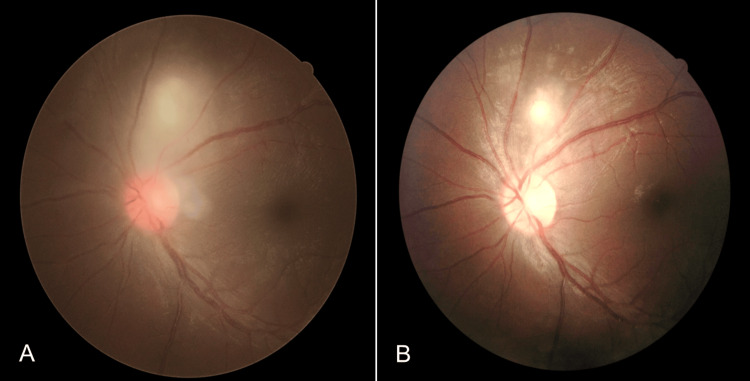
(A): Pre-treatment. (B): Following treatment, the previously seen vitritis in (A) was completely resolved while the focal chorioretinitis lesion became more well-defined

Blood investigations showed a high erythrocyte sedimentation rate (ESR) of 35 mm/hour but serum anti-nuclear antibody and complements 3 and 4 were negative. There were no signs of tuberculosis (Tb) infection in the Mantoux test, sputum Tb workout, and chest X-ray. A presumptive diagnosis of acute panuveitis secondary to OT was made by a medical retina consultant. Thus, the patient was treated with oral Bactrim (sulfamethoxazole 800 mg/trimethoprim 160 mg) per day for 8 weeks and topical corticosteroid (Gutt. Dexamethasone 0.1%) 4 hourly. Oral prednisolone at 1 mg/kg/dose per day was started after 48 hours of oral Bactrim administration with a gradual tapering dose of 5 mg/week for 8 weeks. OT was later confirmed when enzyme-linked immunosorbent assay (ELISA) for serum toxoplasmosis immunoglobulin M (IgM) antibody sent earlier came back as positive. In the fourth week of treatment, left focal retinitis became more well-defined and vitritis was resolved (Figure 4B).

**Figure 5:**
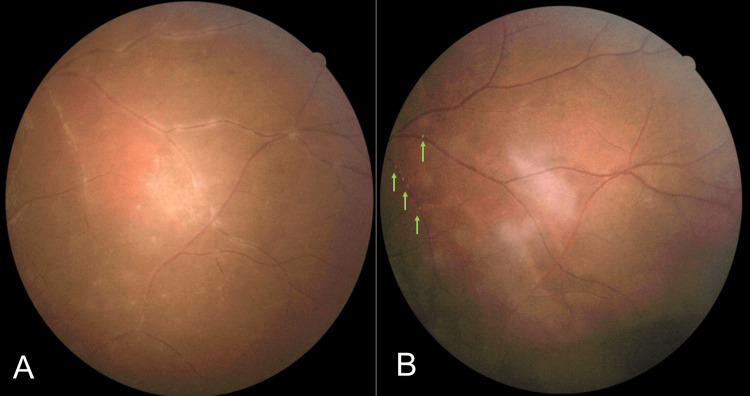
(A): Pre-treatment. (B): Multiple punctate inferior choroiditis were resolved and resolving peripheral vasculitis along with Kyrieleis plaques (green arrows) were observed during the treatment (B)

The remote peripheral vasculitis and multiple inferior punctate choroiditis were resolved but the presence of Kyrieleis plaques was noted during the fourth week of follow-up (Figure 5B). At two months post-treatment, all the presented clinical signs were resolved and the left eye's visual acuity improved to 6/6.

## Discussion

OT patients usually complain of floaters, blurring of vision, or vision loss. The causes of presented visual symptoms in OT is due to the recurrent posterior uveitis [5]. Typical OT is marked by the presence of both focal, white, large, full thickness necrotizing retinochoroiditis and severe vitritis manifesting as the classic sign of “headlight in the fog” [6]. It usually occurs unilaterally, adjacent to a pigmented retinochoroidal scar from a previous active toxoplasmosis infection, and is associated with retinal vasculitis [7].

Atypical OT encompasses multifocal retinochoroiditis, mild or absent vitritis, an active lesion with the absence of a chorioretinal scar (as in this case), bilateral eye involvement, papillitis, choroiditis without retinitis, hemorrhagic vasculitis, retinal artery occlusion, neovascularization, and retinal detachment [7-9]. During the acute stage of infection, retinal vasculitis in typical OT is usually seen at the local venous vasculature surrounding the retinochoroiditis area [10]. However, 36% of atypical vasculitis may also occur remotely from the active lesion, which involves four quadrants or diffuse vasculitis as in this case presentation [10,11].

Kyrieleis arteritis, also known as segmental retinal arteritis (SRA), is rare to be seen in posterior uveitis, however, it is most often reported in OT [12]. SRA indicates a severe inflammation but its presence does not worsen its prognosis [13]. White-yellowish exudates, also known as Kyrieleis plaques, are present in a beaded pattern that does not extend beyond the thickness of the artery [14]. Kyrieleis plaques were observed in this case at the remote vasculitis site during treatment as the vitritis and choroiditis had resolved.

About 45% of retinochoroiditis with retinal vasculitis were presumptively diagnosed OT [14]. Almost all serology confirmed that OT with retinochoroiditis near a pigmented scar had retinal vascular involvement ie: perivenous sheathing, periarterial sheathing, and focal periarterial plaques without leakage (Kyrieleis arteritis) [14]. Atypical OT can be challenging in its diagnosis and requires a serology antibody to *T. gondii *(IgM/ IgG) or polymerase chain reaction (PCR) test for aqueous or vitreous samples in detecting the *T. gondii* organism [15]. However, ELISA remains a widely used method for its exceptional sensitivity and specificity in detecting the *T. gondii* antibody and its antigenically active substances dependent on the availability of hospital laboratory facilities [16].

Classic medical therapy in acute toxoplasmosis retinochoroiditis involves a combination of anti-microbial (eg. pyrimethamine and sulphonamides) and steroids for a period of four to six weeks [17,18]. Alternative antimicrobials, such as sulfamethoxazole-trimethoprim, are popular for being well tolerated by patients and their low cost and wide availability, and are as effective as the classic therapy in reducing lesion size [18,19].

Over half of OT patients with variable periods of follow-up had a recurrence of active retinitis in a mean of 5.8 years [6]. In immunocompetent individuals, retinitis typically follows a self-limiting course and tends to resolve completely within 30 to 60 days, even without any treatment [4,18]. The severity of visual loss is dependent on the site of the chorioretinitis. OT patients may still have poor vision despite fully recovering from active OT if the chorioretinal scar involves the optic disc or macula. Complications of OT include isolated retinal tear (6%), retinal detachment (6%), retinal vascular occlusion (4.5%), epiretinal membrane (7%), macular edema (12%), subretinal neovascularization (4%), cataract (13%), and optic nerve atrophy (4%) [20].

## Conclusions

This case report highlighted the atypical features of OT with the absence of a chorioretinal scar and vasculitis adjacent to the focal chorioretinitis area. The atypical remote localization of vasculitis and choroiditis at the far periphery retina in the classic appearance of “headlight in the fog” chorioretinitis is strongly due to toxoplasmosis. A Kyrieleis plaque is rarely seen, and it is important to recognize its indication (severity of the inflammation). Autoimmune and other infective causes need to be ruled out to identify the culprit of acute panuveitis. Early initiation of antibiotic therapy is warranted based on the suggestive clinical signs despite pending a confirmation serology test, to ensure a good final visual and ocular outcome. Topical and oral steroids should be administrated concomitantly with antibiotics in order to reduce the ongoing inflammations and prevent further damage to the ocular structures. Further research on this atypical OT with remote vasculitis and its clinical implications should be conducted to enhance our understanding of its pathophysiology and aid in early management.
